# Transcriptional profiles associated with coronary artery disease in type 2 diabetes mellitus

**DOI:** 10.3389/fendo.2024.1323168

**Published:** 2024-04-19

**Authors:** Jose B. Nevado, Eva Maria C. Cutiongco-de la Paz, Elizabeth T. Paz-Pacheco, Gabriel V. Jasul, Aimee Yvonne Criselle L. Aman, Christian Deo T. Deguit, Jana Victoria B. San Pedro, Mark David G. Francisco

**Affiliations:** ^1^ Institute of Human Genetics, National Institutes of Health-University of the Philippines Manila, Manila, Philippines; ^2^ Philippine Genome Center, University of the Philippines System, Diliman, Quezon City, Philippines; ^3^ Division of Endocrinology, Department of Medicine, University of the Philippines-Philippine General Hospital Medical Center, Manila, Philippines

**Keywords:** type 2 diabetes mellitus, coronary artery disease, transcriptomics, differential gene expression, pathway analysis

## Abstract

**Background:**

Coronary artery disease (CAD) is a common complication of Type 2 diabetes mellitus (T2DM). Understanding the pathogenesis of this complication is essential in both diagnosis and management. Thus, this study aimed to characterize the presence of CAD in T2DM using molecular markers and pathway analyses.

**Methods:**

The study is a sex- and age-frequency matched case-control design comparing 23 unrelated adult Filipinos with T2DM-CAD to 23 controls (DM with CAD). Healthy controls served as a reference. Total RNA from peripheral blood mononuclear cells (PBMCs) underwent whole transcriptomic profiling using the Illumina HumanHT-12 v4.0 expression beadchip. Differential gene expression with gene ontogeny analyses was performed, with supporting correlational analyses using weighted correlation network analysis (WGCNA).

**Results:**

The study observed that 458 genes were differentially expressed between T2DM with and without CAD (FDR<0.05). The 5 top genes the transcription factor 3 (*TCF3*), allograft inflammatory factor 1 (*AIF1*), nuclear factor, interleukin 3 regulated (*NFIL3*), paired immunoglobulin-like type 2 receptor alpha (*PILRA*), and cytoskeleton-associated protein 4 (*CKAP4*) with AUCs >89%. Pathway analyses show differences in innate immunity activity, which centers on the myelocytic (neutrophilic/monocytic) theme. SNP-module analyses point to a possible causal dysfunction in innate immunity that triggers the CAD injury in T2DM.

**Conclusion:**

The study findings indicate the involvement of innate immunity in the development of T2DM-CAD, and potential immunity markers can reflect the occurrence of this injury. Further studies can verify the mechanistic hypothesis and use of the markers.

## 1 Introduction

It is widely accepted that Type 2 diabetes mellitus (T2DM) increases the likelihood of developing coronary artery disease (CAD) (1), which leads to a medical condition termed diabetic CAD (DM-CAD). Separately, T2DM and CAD are significant global concerns affecting individuals of all ethnicities and geographic locations (2). Unfortunately, the interaction between T2DM and CAD results in excessive morbidity and mortality, wherein 75% of T2DM patients die due to CAD.

Despite the advancements in intervention devices and medications, T2DM-CAD remains a clinical conundrum. This warrants further investigation of its molecular mechanisms that can yield novel perspectives that may improve the management of T2DM CAD. As people with diabetes have different susceptibility to CAD, several studies have shown genetic differences (3, 4). This is important as pathogenesis and subsequent management diverge significantly from other T2DM patients and CAD-related injuries due to other causes, such as primary hypertension or degenerative changes (2, 5). DM-related CAD usually manifests early in life, involving diffuse lesions with more involved major coronary vessels. In a recent study, for example, blood triglyceride levels (6) and nutritional interventions (7) seem to affect DM-CAD more than nonDM-CAD in terms of clinical outcomes. This finding implies distinct mechanisms for DM-CAD compared to other risk factors.

Gene expression profiling, in particular, provided insights into which molecular pathways had been linked to the development of certain diseases, although recently more into oncology and drug discovery. However, most gene expression studies for T2DM only address the disease’s presence and rarely the specific complications. In addition to the mechanistic insights, whole transcriptome studies can lead to discoveries of molecular markers that can impact the diagnosis, prognosis, and monitoring of complications.

By performing whole transcriptome interrogation, the present study aimed to surmise molecular mechanisms associated with CAD in T2D, infer potential causal mechanisms, and determine molecular markers associated with the condition.

## 2 Materials and methods

### 2.1 Study design and patient recruitment

The study was implemented from March 2014 to December 2018 upon the approval of the Review Ethics Board of the University of the Philippines Manila (UPMREB-2012-0184-NIH). The study used an age- (within three years) and sex- frequency-matched case-control design comparing participants with T2DM with coronary artery disease to those without CAD. A reference comparator comprised a healthy control set without T2DM. Upon signing an informed consent from the Philippine General Hospital Medical Center, the study participants were enrolled in primary care clinics, communities, and hospitals within Metro Manila.

### 2.2 Inclusion and exclusion criteria

The main comparators will comprise adult (>18-year-old) participants with Filipino lineage up to the 3^rd^ generation of ascendancy and are not related to at least three generations of consanguinity. There should be documented T2DM that satisfies the following criteria according to the American Diabetes Association (8): fasting blood sugar (FBS) ≥ 126 mg/dL or glycated hemoglobin (HbA1C) ≥ 6.5% or random blood sugar (RBS) ≥ 200 mg/dL with signs and symptoms of hyperglycemia or 2-hour post-75-gram oral anhydrous glucose tolerance test (2-hr. OGTT) ≥ 200 mg/dL.

The non-T2DM reference group is defined by the following criteria for non-diabetics by the presence of all the following: FBS < 100 mg/dL (5.6 mmol/L), 2-hr OGTT <140 mg/dL, HbA1c <6.5%, and no family history of diabetes mellitus within first-degree relatives. The reference group was age- and sex-matched with the comparator groups.

CAD was determined clinically by the presence of any of the following prior to or concurrent with the first diagnosis of T2DM: documented myocardial infarction (MI), typical angina (described to have at least one of the following symptoms based on the ACC/AHA/ACP-ASIM guidelines (9): retrosternal chest discomfort; localized pain in the epigastrium, back, neck, jaw, or shoulders; pain precipitated by exertion, eating, exposure to cold, or emotional stress, lasting for about 1-5 minutes and relieved by rest or nitroglycerin; or pain intensity that does not change with respiration, cough, or change in position); atypical angina with positive stress test; angiographic evidence of stenosis involving at least one large epicardial artery; or documented history of coronary artery revascularization or reperfusion therapy. Non-CAD controls should not have any of the above signs and symptoms despite having documented T2DM in the past 15 years.

The exclusion criteria include previous diagnosis with type 1 diabetes mellitus, current pregnancy or lactation, active alcohol abuse or illicit drug use within three months, malignancy with active systemic disease, and disease-free malignancy for less than five years.

### 2.3 Clinical data collection

If applicable, demographic data and clinical characteristics (diagnosis, comorbidities, medications taken, and available laboratory results) were obtained through interview and chart review. Laboratory tests and imaging include lipid profile (triglyceride, high-density lipoprotein, low-density lipoprotein, serum creatinine, aspartate aminotransferase, alanine transaminase, alkaline phosphatase, urine albumin-creatinine ratio, 12-lead electrocardiogram, fundoscopy, and ankle-brachial index.

### 2.4 Sample collection and processing

Within 2 hours of blood collection, peripheral blood mononuclear cells (PBMCs) from 4-ml blood samples were isolated using density gradient centrifugation through Lymphoprep (Stemcell Technologies, Vancouver, Canada). Using 1:1 dilution, the mixture was centrifuged at 800 g for 20 minutes at room temperature, with the Lymphoprep-plasma interphase retained, washed, and pelleted. Isolated PBMCs were resuspended in 0.5 ml Trizol, and temporarily stored at -80°C until RNA extraction, which is two weeks within the extraction. Total RNA was extracted from the PBMCs using QIAamp RNA Blood Mini Kit (Qiagen, Hilden, Germany) following the manufacturer’s instructions. Quantification and OD260/280 determination of total RNA from PBMCs were performed using a NanoDrop 1000 spectrophotometer (Thermo Fisher Scientific, Waltham, MA, USA). The quality of the total RNA in terms of RNA Integrity Number (RIN) was analyzed on the 2200 TapeStation (Agilent Technologies, Santa Clara, CA) system using the TapeStation RNA analysis kits. Typically, RIN scores remain ≥ 6 upon microarray processing, but some samples were not run due to the unavailability of tapes. All RNA samples had A260:280 at 2.00 to 2.20 before microarray processing.

Various studies have indicated that high-quality RNA samples can be extracted from PBMC samples stored at -80°C, even in different media (10–12), even up to 83 days from blood extraction (13). Data for Qiazol-resuspended PBMCs showed that quality is retained (RIN>7) regardless of whether the samples were stored in an RNA preservation agent or not.

### 2.5 Whole-genome expression profiling and microarray data processing

Whole genome gene expression profiling was performed following manufacturers’ protocols of two microarray platforms: (1) the Illumina DirectHyb HumanHT-12 v4.0 Expression Beadchip (Illumina, San Diego, CA, USA) comprising 47,231 oligonucleotide probes representing 34,602 genes and scanned on the Illumina HiScan System (RRID : SCR_020126). The raw gene expression data and initial pre-processing were acquired via GenomeStudio Software version 2011.1 (Illumina; RRID : SCR_010973). Data were normalized using quantile normalization.

All subsequent data processing and integration procedures were performed using R statistical programming language (version 3.5.1). To ensure uniformity of raw data pre-processing procedures between the two different platforms for each analysis, raw gene expression values from the Illumina platform were forced to be positive through an offset via the *lumi* package, subjected to quantile normalization using the *preprocessCore* package and log-transformed via the *Biobase* package and R default functions. Collapsing of probes with multiple gene targets and inter-platform normalization (i.e., removal of batch effects and preservation of variance due to affliction through designated “Batch” and “Affliction” parameter values for each sample) were performed using weighted gene correlation network analysis (*WGCNA)* (14) and the linear models for microarray data (*limma*) packages (15).

### 2.6 Differential gene expression

The gene expression datasets for the study were analyzed using the *limma* package in R [15; RRID : SCR_001905], explicitly computing for empirical Bayes t-statistics, B-statistics, and log2-fold change (logFC) to determine significant differences in gene expression. Differentially expressed genes were determined and ranked according to the Benjamini-Hochberg adjusted p-value with a set cutoff 0.05.

Overlapped differentially expressed genes (DEGs) were also determined in participants with T2DM with CAD compared with T2DM without CAD and healthy controls. The results were illustrated using the Venn diagram web tool of the VIB-UGent Center for Plant Systems Biology (http://bioinformatics.psb.ugent.be/webtools/Venn/; RRID : SCR_002083).

Supplemental statistical analysis was done using GraphPad Prism (version 8.4.2; GraphPad Software, La Jolla, CA, USA; RRID : SCR_002798) and STATA v14.2 (Stata Corp., College Station, TX; RRID : SCR_012763). Differences within multiple groups were analyzed using the non-parametric Kruskal-Wallis H test and *posthoc* analysis using Dunn’s multiple comparisons tests. Differences between the two groups were analyzed using the non-parametric Wilcoxon signed-rank test for paired data. In contrast, the non-parametric Mann-Whitney U test was used when comparing unpaired data. Finally, receiver operating characteristic curve (ROC) analysis evaluated the diagnostic potential of identified genes of interest, with genes with an area-under-the-curve (AUC) of >0.80 being considered to have favorable ROC statistics.

### 2.7 Weighted gene co-expression network analysis

Weighted gene co-expression network analysis (WGCNA, RRID : SCR_003302) was performed separately on the gene expression dataset using the *WGCNA* package in R (16). For each study, a scale-free topology network of genes was constructed by calculating the biweight mid-correlation between different pairs of genes in all samples (16). To calculate the adjacency matrix, the soft-thresholding power β of 8 was applied. The adjacency matrix was transformed into a topological overlap matrix, from which the dissimilarity values were used to construct the scale-free topology network. Gene modules from the resulting network were produced by setting the minimum module size to 30. Gene modules labeled with arbitrary colors were tested for association with CAD, complications of interest, and comorbidities based on the correlation between the module eigengenes and the traits mentioned above.

To facilitate a concise discussion of the findings for the gene modules, we will only be extensively discussing modules deemed to be of interest (from now on referred to as “top modules”). The top 5 modules of interest will be selected if they strongly correlate with the condition and are not significantly correlated with potential confounders such as age and sex. This will ensure that the gene modules are purely associated with the disease condition and not affected by the inherent biology of the individuals.

### 2.8 Gene ontogeny analysis

Candidate genes from the DGE overlap analysis and significant modules from WGCNA were characterized via functional enrichment analysis to identify likely molecular mechanisms. Gene lists were uploaded to the STRING 12.0 database (Search Tool for the Retrieval of Interacting Genes/Proteins; https://string-db.org; RRID : SCR_005223). A minimum required interaction score threshold of 0.7 for the significant modules was applied. Biological processes that the gene modules could be involved in were identified.

### 2.9 Module-SNP causation inference

To explore the potential causality of the modules, we utilized the findings of a related genotyping study within the research program in this project is included (unpublished results). The genotyping study aimed to identify single nucleotide polymorphisms (SNPs) associated with the same conditions, mainly utilizing the same recruited participants. With this analysis (from now on referred to as “module-SNP causation inference”), the potential causality of the module with the specific condition was deemed if several SNPs were commonly found enriched in a specific module.

## 3 Results and discussion

Understanding the pathogenesis of coronary artery disease (CAD) in type 2 diabetes mellitus (T2DM) is crucial in improving its clinical management, including treatment, diagnosis, and prevention. Thus, this study attempted to determine gene biomarkers and biological processes associated with this condition. It compared a group of T2DM with CAD (DMCAD, n=23) with two comparator groups of age- and sex-matched participants of T2DM without CAD (DMNoCAD, n=23) and without T2DM and no CAD (NoDMNoCAD, n=23). Due to the chronic nature of both T2DM and CAD and the inflammatory assumptions for atherosclerosis and CAD, the study used peripheral blood mononuclear cells (PBMCs) to reflect likely causal mechanisms and involved DM with a minimum of 15 years in duration.

### 3.1 Clinical data

**Table 1** summarizes the clinical characteristics of the participants of this study. The mean age and sex frequency values of all the clinical groups were comparable. Expectedly, both diabetic groups exhibited a higher proportion of hypertension (p<0.001) and metabolic syndrome (p=0.036) compared with healthy individuals. LDL levels (p=0.012) were reduced in both diabetic groups, probably due to the intake of hypolipidemic agents. Glycated hemoglobin (HbA1c) levels were higher among people with diabetes but were not significantly different among the diabetic groups.

**Table 1:** Clinical characteristics of participants of the CAD study.

	DM CAD	DMNoCAD	NoDMNoCAD	p value
General data				
Age (years), mean ± SD	61.39 ± 7.70	61.30 ± 6.70	59.70 ± 7.19	ns
Male sex, frequency (%)	8 (34.8)	8 (34.8)	8 (34.8)	ns
Body mass index, mean ± SD	24.73 ± 3.37	24.99 ± 4.47	23.80 ± 6.67	ns
Currently smoking	2 (8.7, 23)	1 (4.6, 22)	1 (4.8, 21)	ns
Laboratory parameters				
HbA1c (%), mean ± SD	8.60 ± 1.99^a^	8.97 ± 1.90^b^	5.61 ± 0.27^c^	<0.001 (a,b>c)
Triglyceride (mg/dL), mean ± SD	146.47 ± 73.10	128.08 ± 59.08	99.45 ± 48.29	ns
HDL-C (mg/dL), mean ± SD	48.95 ± 12.87	51.40 ± 13.80	53.74 ± 14.43	ns
LDL-C (mg/dL), mean ± SD	117.39 ± 36.76^a^	117.93 ± 46.58^b^	148.47 ± 34.62^c^	0.012 (a,b<c)
Co-morbidities n (%, N)				
Obesity	3 (13.0, 23)	2 (8.7, 23)	6 (27.3, 22)	ns
Hypertension	22 (96.6, 23)a	21 (91.3, 23)b	12 (52.2, 23)c	<0.001
Metabolic syndrome	17 (73.9, 23)a	17 (73.9, 23)b	8 (40.0, 20)c	0.036
Diabetic complications, n (%, n)				
Nephropathy	6 (37.5, 16)	11 (57.9, 19)	-	ns
Retinopathy	10 (66.7, 15)	13 (65.7, 20)	-	ns
Peripheral arterial disease	8 (36.4, 22)	9 (42.9, 21)	-	ns
Cerebrovascular disease	6 (27.3, 22)	2 (8.7, 23)	-	ns

ns, not significant; SD, standard deviation.

A comparison with a CAD-only group is interesting in inferring differences with respect to the presence of T2DM, but is not the aim of the paper. However, such an approach may not be appropriate as the group is from a different source population, which is not the interest of the study. Nonetheless, the comparison to a CAD-only group might be interesting in future investigations. As a main principle in a case-control-designed study, the comparators must come from a common source population (17, 18). In this paper, the assumed source population is composed of subjects with T2DM, of which all subjects were selected. In effect, the assumption is that CAD occurs in the context of T2DM. The healthy group served as a reference for the normal gene expression (down- or upregulation).

### 3.2 Differentially expressed genes in T2M-CAD

**Figure 1:**
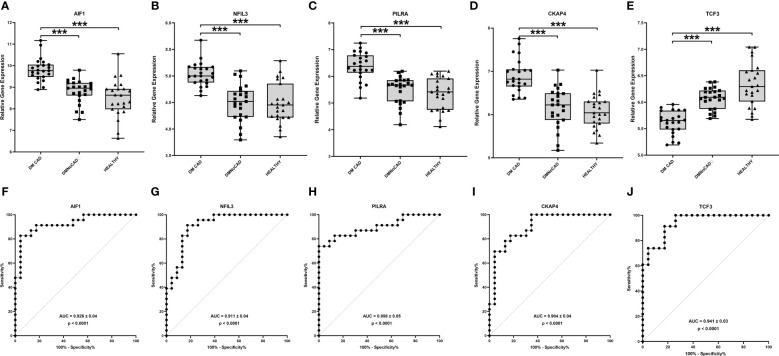
TCF3, AIF1, NFIL3, PILRA and CKAP4 are potential biomarkers for T2DM CAD. In T2DM with CAD, expression levels of TCF3 **(A, F)** were higher while that of AIF1 **(B, G)**, NFIL3 **(C, H)**, PILRA **(D, I)** and CKAP4 **(E, J)** were lower compared with T2DM without CAD and with healthy controls. TCF3 displayed the highest diagnostic potential with the largest ROC-AUC in differentiating diabetic individuals with and without CAD. Significant differences in gene expression were determined through Kruskal Wallis test with posthoc Dunn’s multiple comparisons test: ***p<0.001.

Using differential gene expression analyses of PBMCs, 458 genes were differentially expressed between T2DM with and without CAD (Benjamini-Hochberg adjusted p value<0.05). For consideration for diagnostic marking, we presented the 5 top genes according to p-value (**Table 2** and **Figure 1**). These include the *transcription factor 3* (*TCF3*), *allograft inflammatory factor 1* (*AIF1*), nuclear factor, interleukin 3 regulated (*NFIL3*), *paired immunoglobulin-like type 2 receptor alpha* (*PILRA*), and *cytoskeleton-associated protein 4* (*CKAP4*). The gene expression levels of the top genes in the various groups were illustrated in **Figures 1A–E** with the corresponding receiver-operator curve analyses (**Figures 1F–J**). In DMCAD, expression levels of *TCF3* (A, F) were lower while that of *AIF1* (B, G), *NFIL3* (C, H), *PILRA* (D, I), and *CKAP4* (E, J) were higher compared with DMNoCAD and with NoDMNoCAD. Among the selected genes, *TCF3* has the highest AUC (0.941 ± 0.03). Hence, it is interesting that the genes retained a solid statistical association that may warrant further research as potential biomarkers for diabetic CAD.

**Table 2:** Top differentially expressed genes in T2DM with CAD versus those without CAD.

Gene symbol	Description	Relevant function	Fold change (log 2)	Adjusted p-value
*TCF3*	transcription factor 3	Member of the E protein (class I) family of helix-loop-helix transcription factors	-0.458	1.117E-04
*AIF1*	allograft inflammatory factor 1	The protein binds actin and calcium. Plays a role in vascular inflammation	1.005	1.603E-04
*NFIL3*	nuclear factor, interleukin 3 regulated	The transcriptional regulator that binds as a homodimer to activating transcription factor (ATF) sites in many cellular and viral promoters	0.534	3.449E-04
*PILRA*	paired immunoglobin-like type 2 receptor alpha	ITIM-bearing member of the paired immunoglobin-like type 2 receptor pair, which functions in the inhibitory role. Involved in cell signaling	0.890	3.449E-04
*CKAP4*	cytoskeleton-associated protein 4	High-affinity epithelial cell surface receptor for antiproliferative factor, which mediates the anchoring of the endoplasmic reticulum to microtubules	0.736	3.449E-04

To emphasize, the basis for the selection of the five expression markers is the p values based on the Benjamini-Hochberg multiple testing adjustment. These genes are just arbitrary representations to demonstrate the possibility of transcriptional marking, as well as secondary mechanistic inferences. In effect, the paper still recognized the whole transcriptome in its analyses to infer its main mechanistic insights.

The idea behind differential gene expression analyses of whole transcriptomes is that genes that are statistically different in expression be identified and interpreted biologically. Therefore, it is possible that genes that are typically expressed in PBMCs but are not differentially expressed will not come out as interesting. The findings of the top genes being not obviously related to immunity or inflammation make the present findings intriguing and suggest novel insights into the role of PBMCs in T2DM-associated CAD.

Taken together, the top genes still suggest an involvement of the immune response and reparative processes with the development of atherosclerosis, which may eventually progress to coronary artery disease. However, it has not yet been established how directly these genes are related to diabetic CAD, with causality being speculative and circumstantial. Nonetheless, the results are limited to the expression of the PBMCs, and data is biased toward immunity.

*TCF3* has been reported to be a marker for CAD. It is best known to be the downstream repressor for the Wnt signaling implicated in cellular proliferation during development and repair processes (19). In particular, its involvement in intracellular signaling for T-cell development and its downregulation has been implicated (20). However, the role of *TCF3* in B cell development is well known (21). its role in CAD seems to be a good point of interest.

*AIF1* expression has been proposed as an indicator of activated macrophages (22). Cytokines and interferon activate it and may enhance macrophage activation and growth of blood vessel smooth muscle cells and T-lymphocytes. Macrophage activation is a critical step in the development and progression of inflammation, and macrophages are a primary biological component of atherosclerotic plaque and are present at all phases of atherogenesis, the process of atherosclerotic plaque formation that leads to CAD (23). *In vivo*, *AIF1* is located in the same area as CD68+ macrophages in human arteries affected by atherosclerosis (24). Additionally, mice genetically modified to overproduce *AIF1* in microparticles (25) or vascular smooth muscle cells (26) showed an increased risk of atherosclerosis. Shirai et al. (27) also uncovered the possibility that macrophages and elevated glucose uptake are related. Accordingly, macrophages ensconced in an arterial plaque displayed an increased glucose uptake that could cause the heart to become hyper-inflammatory. These findings imply that CAD may partly be induced by hyper-aggressive macrophages that gorge on glucose.

*NFIL3*, which is also referred to as the b-ZIP transcription repressor, E4-binding protein 4 (E4BP4), a protein that functions as a transcription regulator, which binds to activating transcription factor (ATF) sites found in various cellular and viral promoters as a homodimer. The expression of E4BP4 is primarily found in the liver and is just slightly present in the heart, lung, brain, spleen, and skeletal muscle of mice (28). What function E4BP4 performs in the heart, however, remains uncertain. Although no published studies explicitly associate E4BP4 with T2DM CAD, it was implicated in CAD. In a 2017 study that integrated five datasets of coronary heart disease, E4BP4 was among the top genes that were differentially expressed between patients and controls (29). These results indicate that E4BP4 may play a substantial role in coronary heart disease. Remarkably, as a protein wearing multiple hats, E4BP4 also plays a significant role in innate immunity, which is presumed to be linked to the development of CAD (30, 31). E4BP4 is required to grow NK cells and CD8a+ traditional dendritic cells. It also plays a role in macrophage activation, the polarization of CD4+ T cell responses, and B cell class switching to IgE, all of which can exacerbate or mitigate the atherogenic process and the onset of CAD.

Although the role of *PILRα* in diabetic CAD has not yet been established, it has been associated with immune activation. The PILRα gene, a cell surface receptor that detects particular O-glycosylated proteins, is present in various innate immune cells. In one study, it is implicated in the negative activation of neutrophils during inflammatory reactions (32) and in monocytic deposition (33). Emerging evidence suggests that neutrophils have a role in sterile inflammation, such as the formation of atherosclerosis, which contributes to CAD (34, 35). Neutrophils that have migrated out of blood vessels release a protein called Cramp, carried back across the endothelium, activating a receptor called formyl-peptide receptor 2, which subsequently activates a protein called β1/2 integrin and recruits inflammatory monocytes. This lends credence to the notion that neutrophils contribute to the emergence of inflammatory diseases (35). Moreover, PILRα is essential in regulating neutrophil recruitment during inflammatory responses by modulating the activation of integrins induced by chemical attractants (32). The ability of neutrophils to draw and direct inflammatory monocytes to regions of atherosclerosis underlies the probable relationship between neutrophils, their granule proteins, and the emergence of atherosclerosis. This is corroborated by the findings that in mice aorta samples, lowering neutrophil counts results in a decrease in monocytes/macrophages (36). Thus, the role of *PILRα* in downregulating myelocytic activity may confer risk to those with impaired genetic function.

*CKAP4* (cytoskeleton-associated protein 4) is a type II transmembrane protein that is present in the endoplasmic reticulum and has been implicated as an interactor with the extracellular matrix and integrins by acting as a receptor for *DKK1* (Dickkopf1) (37). Currently, no evidence shows a direct association between CKAP4 and T2DM-CAD. However, a study discovered that CKAP4 is intimately linked to the progression of atherosclerosis (38). CKAP4 suppresses the recycling of the α5β1 integrin, which is closely associated with atherosclerosis and is essential for the progression of the condition (37, 39). Therefore, *CKAP4* may affect atherosclerosis via regulation of the α5β1 integrin, although further research is required to comprehend these findings fully.

### 3.3 Pathways analyses

#### 3.3.1 Ontogeny analyses for differentially expressed genes

**Figure 2:**
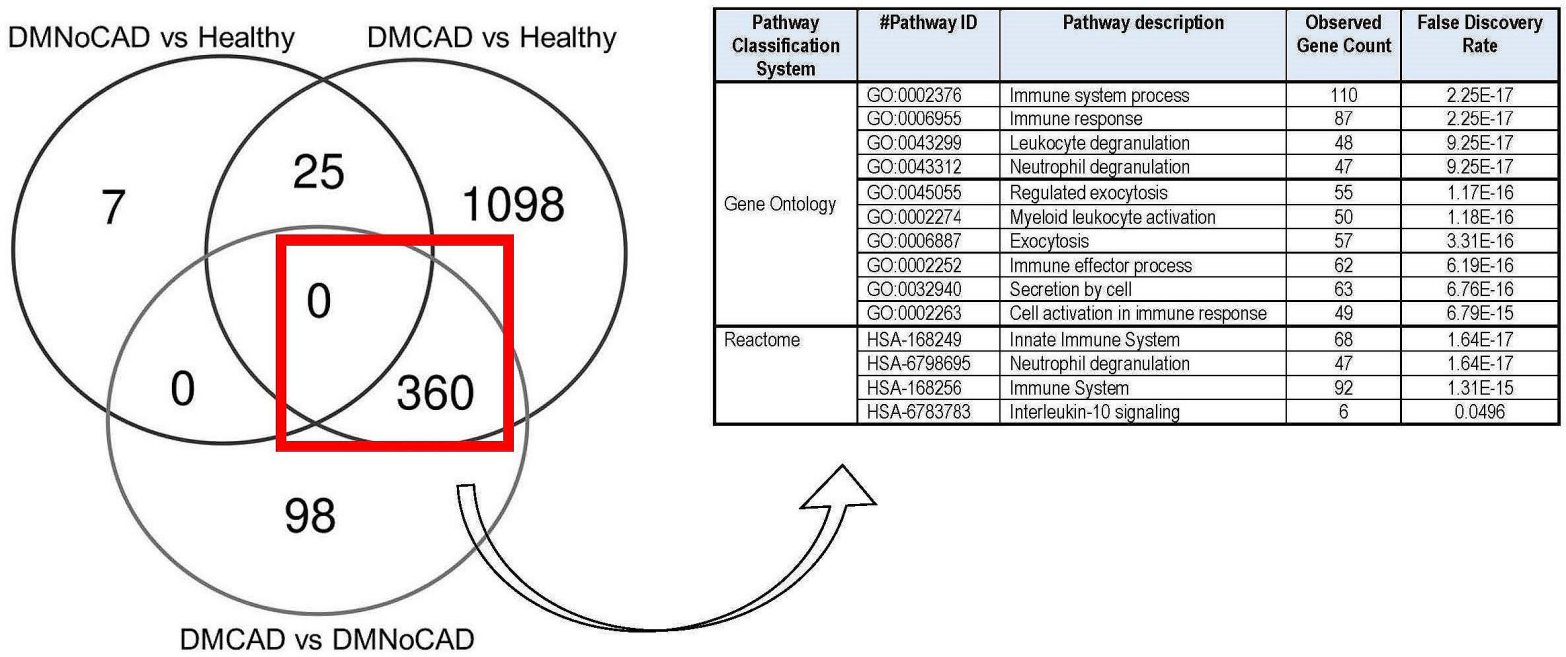
Differential expression of genes involved in immune processes in T2DM CAD. Comparison and gene enrichment of differentially expressed genes (FDR<0.05) common in DMCAD, DMNoCAD, and NoDMNoCAD conditions display potential association of immune system processes with diabetic CAD.

Using overlap analysis of differentially expressed genes from the comparator groups, we showed an enrichment of genes in innate immune system processes. Results show that most genes differentiate CAD from the DM condition (**Figure 2**). Overlapping differentially expressed genes that differentiate CAD from both NoCAD groups (DM and NoDM) revealed a 360-gene set that, on ontogeny analyses, suggests an implied theme on the myeloid and leukocytic component of the immune system. This is consistent with our inference on the role of innate and myelocytic activity in T2DM-CAD.

#### 3.3.2 Weighted gene co-expression network analysis

**Figure 3:**
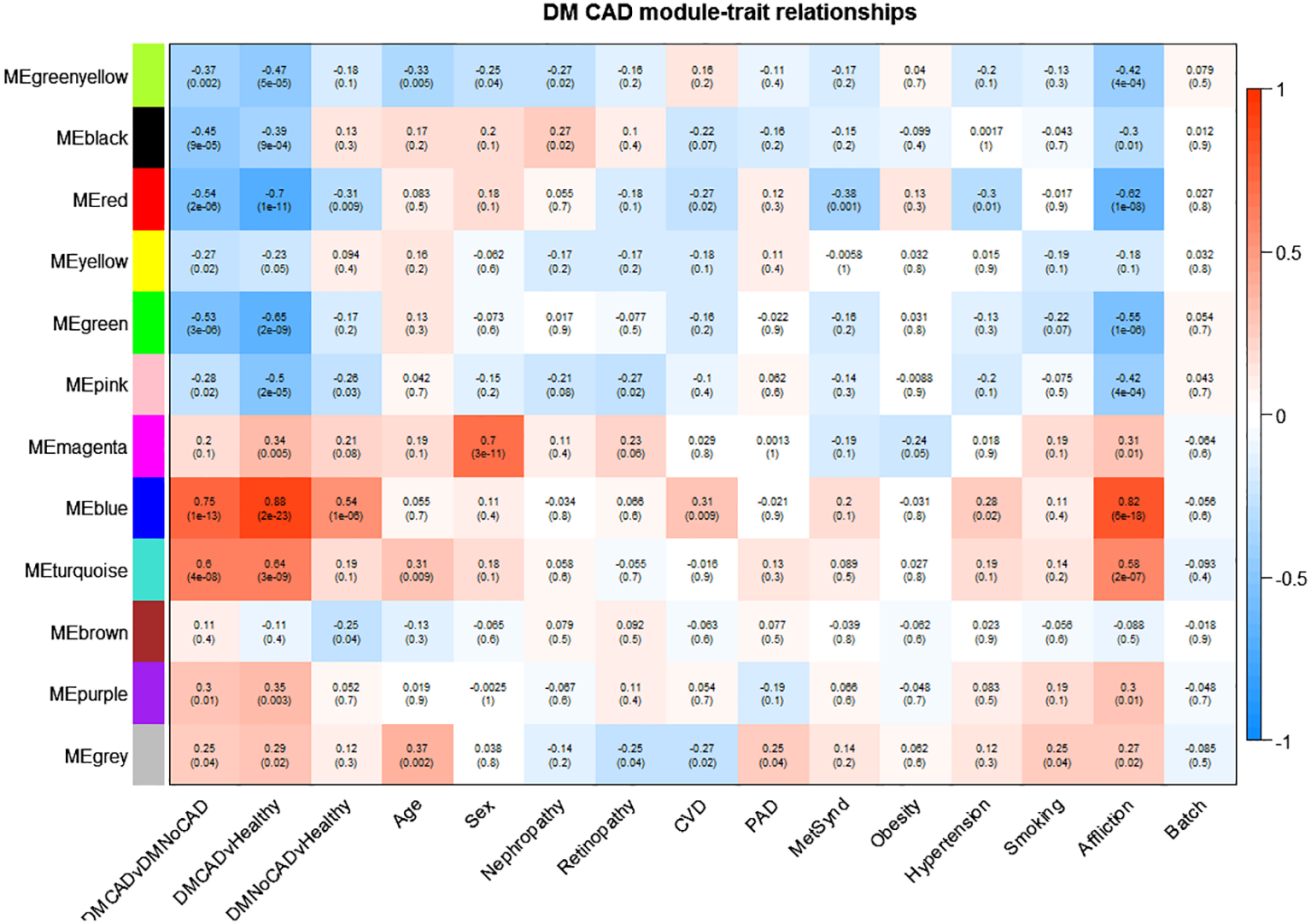
Gene modules correlated with T2DM CAD. T2DM CAD significantly correlates with reduced expression of the black and green modules and increased expression of the turquoise, blue, and purple modules. The black module is enriched for genes responsible for NK cell-mediated cytotoxicity, while the turquoise and blue module is enriched for immune system process-related genes. The purple module is enriched for oxygen transport-genes, while the green module is not enriched for any biological theme. The arrows point to the top modules of interest. Upper values in each box indicate Pearson correlation values (i.e., mean gene significance) while the lower values indicate the corresponding p-value; red shading indicates direct correlation; blue shading indicates inverse correlation).

We perform a weighted gene co-expression network analysis (WGCNA) to support a mechanistic insight further. Results suggest roles for mechanisms involved in NK-cell mediated cytotoxicity (downregulated in CAD; black module) and oxygen transport, probably in conjunction with the immune response (upregulated in CAD; purple module) (**Figure 3** and **Table 3**). Other interesting modules were the green, blue, and turquoise modules. These were discussed in detail below.

**Table 3:** Top enriched biological processes for the genes within the top modules correlated with CAD.

Module	Pathway Classification System	#Pathway ID	Pathway Description	Observed gene count	False Discovery Rate
**Black** **(n=383)**	KEGG	hsa04650	Natural killer cell-mediated cytotoxicity	12	0.0024
**Green** (n=656)	*no significant enrichment*				
**Turquoise** (n=2696)	Gene ontology	GO:0002283	Neutrophil activation is involved in the immune response	115	1.04e-10
	KEGG	hsa04142	Lysosome	32	0.0047
	Reactome	HSA-168256	Immune system	317	2.90e-12
**Black** (n=2562)	Gene ontology	GO:0002376	Immune system process	125	6.29e-10
	KEGG	hsa04380	Osteoclast differentiation	12	0.0399
	Reactome	HSA-168256	Immune system	108	3.52e-11
**Purple** (n=261)	Gene ontology	GO:0015671	Oxygen transport	6	0.00062
		GO:0015701	Bicarbonate transport	6	0.0445
	Reactome	HSA-1247673	Erythrocytes take up oxygen and release carbon dioxide	4	0.0073
		HSA-1237044	Erythrocytes take up carbon dioxide and release oxygen	4	0.0129

The black module was discovered to have genes involved in NK-cell-mediated toxicity. This module showed downregulation in T2DM with CAD and upregulation in T2DM without CAD. A subset of lymphocyte effectors known as NK cells in the innate immune system can attack cells under stress, such as cancerous and virus-infected cells (40). NK cells also release various cytokines and can affect both innate and adaptive immunity (41). Moreover, NK cells may serve a substantial but unclear impact in chronic inflammatory conditions such as atherosclerosis. Previous studies demonstrated a decrease in both the number and activity of NK cells in individuals with CAD when compared to healthy (42, 43). This finding is consistent with reports demonstrating that NK cell numbers diminish in CAD, especially among those with metabolic syndrome (44). This decreased NK cell population could result in reduced activity of inhibitory cytokines, such as NKB1 and CD158b, that can mitigate long-term inflammation (45). On the other hand, apoptosis of NK cells may explain this observation, which is attributed to exposure to toxic oxidized lipids (46, 47). The resolution of the role of NK cells may be crucial in the development of therapies in case of causal implications.

The turquoise module, upregulated in T2DM-CAD, is enriched in themes involved in immune response, specifically neutrophil activation. It has been reported that T2DM patients have higher circulating neutrophil levels than healthy individuals (48). The number of neutrophils in the peripheral blood increases in T2DM and is closely related to the remaining β-cell function. Additionally, experiment data indicate that insulin resistance and high blood sugar levels contribute to increased neutrophil count (49). In addition, there seems to be enhanced systemic and local neutrophil activation in acute coronary syndromes (50).

Notably, the relationship between decreased lymphocyte effector NK-cells and increased expression of neutrophils in T2DM CAD, as demonstrated by the black and turquoise modules, is particularly intriguing. Earlier research has indicated that the neutrophil-to-lymphocyte ratio (NLR) in T2DM patients can predict CAD and coronary artery susceptible plaques (51). Short-term adverse events, such as mortality, coronary artery disease (CAD), stroke, and heart failure, have also been linked to a higher NLR (52). Changes in NLR occur due to a shift in the balance of innate (neutrophils) and adaptive (lymphocytes) immunity. If this proves to be causal, the implication is that interventions targeting this phenomenon may be plausible considerations.

The blue module is also upregulated and correlates significantly with T2DM- CAD. The inferred role of the blue module overlapped with the turquoise module, which centers on the immune system. In line with this, it is already observed that the innate immune system is dysregulated in T2DM (53, 54). It is particularly interesting how the dysregulated immune system mediates the interaction between T2DM and CAD. Interestingly, the current understanding of the pathogenesis of T2DM-CAD focuses on the synergistic interaction between T2DM and CAD through sterile inflammation caused by a dysfunctional immune system (55), which coincides with the thematic role of the blue module.

The purple module was also enriched with oxygen transport themes. In T2DM-CAD, the oxygen transport was upregulated. As this theme was derived from the gene expression of PBMCs, this is probably a response to chronic mitochondrial dysfunction (56) and increased reactive oxygen species production in the blood cells of diabetics (57). Generally, the exact cause of this phenomenon is unclear. Moreover, its cause-effect relationship with CAD needs further investigation.

Lastly, the green module was recognized as significant but contained genes not significantly enriched for any biological process. It has to be noted that since enrichment analysis via STRING is largely based on empirical data, the modules without any significant enrichment could still be representative of a theme that has not been fully elucidated yet.

#### 3.3.3 Genomic variant anchoring analysis

The variants associated with T2DM-CAD from a concurrent related study were identified by genotyping and correlated to the derived modules (see **Table 4**). Hypothetically, the correlation of these variants with the significant modules increases the likelihood of the causal implications of the derived pathways. Here, these associations seem to be enriched in the blue module. As this module appears to be enriched with innate immune response genes, this supports the idea that innate immune system dysfunction may contribute to the development of T2DM-CAD. Reiterating, the blue module, together with the related turquoise module, has been associated with innate immunity — a common theme throughout the paper. This supports the surmised roles of innate immunity, especially of the myelocytic cells, which are repeatedly highlighted. The present data point to this biological process as probably causal for T2DM-associated CAB, which, in turn, could imply potential therapeutic targets in future investigations.

**Table 4:** Association of derived T2DM CAD-associated variants to interesting modules in the study.

Variants*	Implicated Gene	Relevant function	Correlated module/s	Theme description**
rs3755863	*PPARGC1A*	Synonymous/3’ UTR/downstream gene/non-coding transcript exon variant		
rs8192678	*PPARGC1A*	Missense/3’ UTR/downstream gene/non-coding transcript exon variant		
rs1894116	*YAP1*	Intron/regulatory region variant		Platelet degranulation
rs4311394	*ARL15*	Intron variant	Turquoise	Immune system
rs6545814	*ADCY3*	Intron/regulatory region variant	Blue	Immune system process
rs10830963	*MTNR1B*	Intron variant	Green	No significant enrichment
rs7588550	*ERBB4*	Intron/non-coding transcript variant		–
rs4402960	*IGF2BP2*	Intron/non-coding transcript variant		Platelet degranulation
rs1470579	*IGF2BP2*	Intron/non-coding transcript/downstream gene variant		Platelet degranulation
rs2383208	*CDKN2B-* *AS1*	Downstream gene variant	Blue	Immune system process
rs944797	*CDKN2B-* *AS1*	Intron/non-coding transcript/upstream gene/regulatory region variant	Blue	Immune system process

*Variant derived as associated with T2DM CAD at FDR<0.05.

**Associated theme for modules.

This paper underscores the potentially significant role of innate immune reactions in the pathogenesis of CAD in T2DM. The inflammatory inferences of the findings are consistent with the notion that the synergistic interaction between T2DM and CAD can be due to metabolic inflammation or meta-inflammation, a type of inflammation that does not entail an infection (58). The innate immune system is crucial in defending the body against invading pathogens. The downregulation may reflect a dysfunctional state rather than an overactivation. Thus this can also trigger meta-inflammation in DM-CAD (55). Meta-inflammation is caused by an imbalance in the immune system, which can have both positive and negative effects. Meta-inflammation can result in insulin resistance, in which the body’s insulin becomes less effective in its target organs. Typically, meta-inflammation begins as a specific inflammation in organs that are significant targets of insulin, such as adipose tissue, skeletal muscles, and the liver. When the body can no longer manufacture enough insulin to overcome resistance, high blood sugar (hyperglycemia) ensues, leading to T2DM (59). The progression of atherosclerosis is sped up by the hyperglycemia brought on by T2DM, which causes a higher infiltration of inflammatory macrophages and T cells and increases inflammation in the coronary arteries. Meta-inflammation exacerbates the development of atherosclerosis by promoting the buildup of lipids in the arterial walls and stimulating the growth and movement of smooth muscle cells (60).

In exploring whole transcriptome inference, confidence in data interpretation can come from stringent statistics, the strength of gene-gene interactions, and coherence with meaningful biological insights. Validation with other expression platforms for this purpose could lessen the useful information (61). Note that the focus of the present study is to gain insights into the mechanisms associated with T2DM-associated CAD, in which current microarray designs have proved to be very reliable.

The need for other expression platform for validation is important for biomarker discovery (62). This is both for technical (e.g., verification, reproducibility, accuracy) and practical reasons (e.g., cost, portability). The discovery of potential diagnostic markers is a secondary finding that is understandably appealing for future research. For this purpose, the data is shared for data mining with possible future marker validation by other parties (GSE250283 in the Gene Expression Omnibus [GEO datasets] in https://www.ncbi.nlm.nih.gov/gds.

## 4 Summary and conclusion

In summary, the present study points to an association of immune mechanisms to the progression of T2DM-CAD. Specifically, variations in the activity of the innate immune system centering on the myelocytic lineage-derived cells can provide a predisposition to T2DM-CAD. Dysfunction in these cells may have been associated previously with atherosclerosis and CAD, especially among diabetics. This study focused on PBMCs, so that the findings may be limited to a larger picture. Nonetheless, the data still hint that T2DM-CAD injuries may be due to inflammatory dysfunction.

## Data availability statement

The data generated and presented in the study are deposited in the GEO Datasets (https://www.ncbi.nlm.nih.gov/gds) repository of National Center for Biotechnology Information (NCBI) with accession number GSE250283.

## Ethics statement

The studies involving humans were approved by Review Ethics Board of the University of the Philippines Manila. The studies were conducted in accordance with the local legislation and institutional requirements. The participants provided their written informed consent to participate in this study.

## Author contributions

JN: Conceptualization, Formal analysis, Funding acquisition, Investigation, Methodology, Project administration, Resources, Software, Supervision, Visualization, Writing – original draft, Writing – review & editing. EC-dP: Conceptualization, Funding acquisition, Investigation, Methodology, Resources, Supervision, Writing – review & editing. EP-P: Conceptualization, Data curation, Funding acquisition, Investigation, Methodology, Project administration, Resources, Supervision, Writing – review & editing. GJ: Data curation, Investigation, Methodology, Resources, Writing – review & editing. AA: Formal analysis, Software, Visualization, Writing – review & editing. CD: Formal analysis, Software, Visualization, Writing – review & editing. JS: Visualization, Writing – original draft, Writing – review & editing. MF: Data curation, Investigation, Supervision, Writing – review & editing.

## References

[1] Grant PJ Cosentino F Marx N. Diabetes and coronary artery disease: not just a risk factor. Heart (British Cardiac Society). (2020) 106:1357–64. doi: 10.1136/heartjnl-2019-316243 32499237

[2] Aronson D Edelman ER. Coronary artery disease and diabetes mellitus. Cardiol Clinics. (2014) 32:439–55. doi: 10.1016/j.ccl.2014.04.001 PMC4672945 25091969

[3] Ma RC. Genetics of cardiovascular and renal complications in diabetes. J Diabetes Invest. (2016) 7:139–54. doi: 10.1111/jdi.12391 PMC4773661 27042264

[4] Song Y Choi JE Kwon YJ Chang HJ Kim JO Park DH. Identification of susceptibility loci for cardiovascular disease in adults with hypertension, diabetes, and dyslipidemia. J Trans Med. (2021) 19:85. doi: 10.1186/s12967-021-02751-3 PMC7905883 33632238

[5] Hegde SS Mallesh P Yeli SM Gadad VM Punja. M,G. Comparitive angiographic profile in diabetic and non-diabetic patients with acute coronary syndrome. J Clin Diagn Res JCDR. (2014) 8:MC07–10. doi: 10.7860/JCDR/2014/9072.4851 PMC4225925 25386473

[6] Pan Y Wu TT Mao XF Hou XG Yang Y Deng CJ. Decreased free fatty acid levels associated with adverse clinical outcomes in coronary artery disease patients with type 2 diabetes: findings from the PRACTICE study. Eur J Prev Cardiol. (2023) 30:730–9. doi: 10.1093/eurjpc/zwad073 36912007

[7] Li T Yuan D Wang P Zeng G Jia S Zhang C. Association of prognostic nutritional index level and diabetes status with the prognosis of coronary artery disease: a cohort study. Diabetol Metab syndrome. (2023) 15:58. doi: 10.1186/s13098-023-01019-8 PMC10039549 36966329

[8] American Diabetes Association. Standards of Medical Care in Diabetes—2018 Abridged for Primary Care Providers. Clin Diabetes. (2018) 36(1):14–37. doi: 10.2337/cd17-0119 29382975 PMC5775000

[9] Gibbons RJ Chatterjee K Daley J Douglas JS Fihn SD Gardin JM. ACC/AHA/ACP-ASIM guidelines for the management of patients with chronic stable angina: a report of the American College of Cardiology/American Heart Association Task Force on Practice Guidelines (Committee on Management of Patients With Chronic Stable Angina). J Am Coll Cardiol. (1999) 33:2092–197. doi: 10.1016/s0735-1097(99)00150-3 10362225

[10] Palmirotta R De Marchis ML Ludovici G Leone B Savonarola A Ialongo C. Impact of preanalytical handling and timing for peripheral blood mononuclear cell isolation and RNA studies: the experience of the Interinstitutional Multidisciplinary BioBank (BioBIM). Int J Biol Markers. (2012) 27:e90–8. doi: 10.5301/JBM.2012.9235 22562396

[11] Gautam A Donohue D Hoke A Miller SA Srinivasan S Sowe B. Investigating gene expression profiles of whole blood and peripheral blood mononuclear cells using multiple collection and processing methods. PloS One. (2019) 14:e0225137. doi: 10.1371/journal.pone.0225137 31809517 PMC6897427

[12] Zou C Ji C Zhu Y Liu N Zhang S Peng H. Effects of freezing and rewarming methods on RNA quality of blood samples. Biopreservation biobanking. (2023) 21:176–83. doi: 10.1089/bio.2022.0007 35759420

[13] Rodríguez A Duyvejonck H Van Belleghem JD Gryp T Van Simaey L Vermeulen S. Comparison of procedures for RNA-extraction from peripheral blood mononuclear cells. PloS One. (2020) 15:e0229423. doi: 10.1371/journal.pone.0229423 32084228 PMC7034890

[14] Langfelder P Horvath S. WGCNA: an R package for weighted correlation network analysis. BMC Bioinformatics. (2008) 9:559. doi: 10.1186/1471-2105-9-559 19114008 PMC2631488

[15] Ritchie ME Phipson B Wu D Hu Y Law CW Shi W. limma powers differential expression analyses for RNA-sequencing and microarray studies. Nucleic Acids Res. (2015) 43:e47. doi: 10.1093/nar/gkv007 25605792 PMC4402510

[16] Langfelder P Horvath S. WGCNA: an R package for weighted correlation network analysis. BMC Bioinformatics. (2008) 9:559. doi: 10.1186/1471-2105-9-559 19114008 PMC2631488

[17] Setia MS. Methodology series module 2: case-control studies. Indian J Dermatol. (2016) 61:146–51. doi: 10.4103/0019-5154.177773 PMC4817437 27057012

[18] Tenny S Kerndt CC Hoffman MR. Case-control studies. Treasure Island, Florida (FL): StatPearls Publishing (2024). Available at: https://www.ncbi.nlm.nih.gov/books/NBK448143/. 28846237

[19] Wu CI Hoffman JA Shy BR Ford EM Fuchs E Nguyen H. Function of Wnt/β-catenin in counteracting Tcf3 repression through the Tcf3-β-catenin interaction. Dev (Cambridge England). (2012) 139:2118–29. doi: 10.1242/dev.076067 PMC3357906 22573616

[20] McCaffrey TA Toma I Yang Z Katz R Reiner J Mazhari R. RNA sequencing of blood in coronary artery disease: involvement of regulatory T cell imbalance. BMC Med Genomics. (2021) 14:216. doi: 10.1186/s12920-021-01062-2 34479557 PMC8414682

[21] Laidlaw BJ Cyster JG. Transcriptional regulation of memory B cell differentiation. Nat Rev Immunol. (2021) 21:209–20. doi: 10.1038/s41577-020-00446-2 PMC7538181 33024284

[22] Donovan KM Leidinger MR McQuillen LP Goeken JA Hogan CM Harwani SC. Allograft inflammatory factor 1 as an immunohistochemical marker for macrophages in multiple tissues and laboratory animal species. Comp Med. (2018) 68:341–8. doi: 10.30802/AALAS-CM-18-000017 PMC6200031 30227902

[23] Hansson GK. Inflammation, atherosclerosis, and coronary artery disease. New Engl J Med. (2005) 352:1685–95. doi: 10.1056/NEJMra043430 15843671

[24] Tian Y Kelemen SE Autieri MV. Inhibition of AIF-1 expression by constitutive siRNA expression reduces macrophage migration, proliferation, and signal transduction initiated by atherogenic stimuli. Am J Physiol Cell Physiol. (2006) 290:C1083–91. doi: 10.1152/ajpcell.00381.2005 16291819

[25] Mishima T Iwabuchi K Fujii S Tanaka SY Ogura H Watano-Miyata K. Allograft inflammatory factor-1 augments macrophage phagocytotic activity and accelerates the progression of atherosclerosis in ApoE-/- mice. Int J Mol Med. (2008) 21:181–7. doi: 10.3892/ijmm 18204784

[26] Watano K Iwabuchi K Fujii S Ishimori N Mitsuhashi S Ato M. Allograft inflammatory factor-1 augments production of interleukin-6, -10 and -12 by a mouse macrophage line. Immunology. (2001) 104:307–16. doi: 10.1046/j.1365-2567.2001.01301.x PMC1783316 11722645

[27] Shirai T Nazarewicz RR Wallis BB Yanes RE Watanabe R Hilhorst M. The glycolytic enzyme PKM2 bridges metabolic and inflammatory dysfunction in coronary artery disease. J Exp Med. (2016) 213:337–54. doi: 10.1084/jem.20150900 PMC4813677 26926996

[28] Nishimura Y Tanaka T. Calcium-dependent activation of nuclear factor regulated by interleukin 3/adenovirus E4 promoter-binding protein gene expression by calcineurin/nuclear factor of activated T cells and calcium/calmodulin-dependent protein kinase signaling. J Biol Chem. (2001) 276:19921–8. doi: 10.1074/jbc.M010332200 11262393

[29] Shi Y Yang S Luo M Zhang WD Ke ZP. Systematic analysis of coronary artery disease datasets revealed the potential biomarker and treatment target. Oncotarget. (2017) 8:54583–91. doi: 10.18632/oncotarget.17426 PMC5589605 28903366

[30] Male V Nisoli I Gascoyne DM Brady HJ. E4BP4: an unexpected player in the immune response. Trends Immunol. (2012) 33:98–102. doi: 10.1016/j.it.2011.10.002 22075207

[31] Keniry M Dearth RK Persans M Parsons R. New frontiers for the NFIL3 bZIP transcription factor in cancer, metabolism and beyond. Discoveries (Craiova Romania). (2014) 2:e15. doi: 10.15190/d.2014.7 26539561 PMC4629104

[32] Wang J Shiratori I Uehori J Ikawa M Arase H. Neutrophil infiltration during inflammation is regulated by PILRα via modulation of integrin activation. Nat Immunol. (2013) 14:34–40. doi: 10.1038/ni.2456 23142774

[33] Kohyama M Matsuoka S Shida K Sugihara F Aoshi T Kishida K. Monocyte infiltration into obese and fibrilized tissues is regulated by PILRα. Eur J Immunol. (2016) 46:1214–23. doi: 10.1002/eji.201545897 26840635

[34] Döring Y Drechsler M Wantha S Kemmerich K Lievens D Vijayan S. Lack of neutrophil-derived CRAMP reduces atherosclerosis in mice. Circ Res. (2012) 110:1052–6. doi: 10.1161/CIRCRESAHA.112.265868 22394519

[35] Wantha S Alard JE Megens RT van der Does AM Döring Y Drechsler M. Neutrophil-derived cathelicidin promotes adhesion of classical monocytes. Circ Res. (2013) 112:792–801. doi: 10.1161/CIRCRESAHA.112.300666 23283724 PMC3702173

[36] Drechsler M Megens RT van Zandvoort M Weber C Soehnlein O. Hyperlipidemia-triggered neutrophilia promotes early atherosclerosis. Circulation. (2010) 122:1837–45. doi: 10.1161/CIRCULATIONAHA.110.961714 20956207

[37] Osugi Y Fumoto K Kikuchi A. CKAP4 regulates cell migration via the interaction with and recycling of integrin. Mol Cell Biol. (2019) 39:e00073–19. doi: 10.1128/MCB.00073-19 PMC6664606 31160493

[38] Zhu F Zuo L Hu R Wang J Yang Z Qi X. A ten-genes-based diagnostic signature for atherosclerosis. BMC Cardiovasc Disord. (2021) 21:513. doi: 10.1186/s12872-021-02323-9 34688276 PMC8540101

[39] Al-Fakhri N Wilhelm J Hahn M Heidt M Hehrlein FW Endisch AM. Increased expression of disintegrin-metalloproteinases ADAM-15 and ADAM-9 following upregulation of integrins alpha5beta1 and alphavbeta3 in atherosclerosis. J Cell Biochem. (2003) 89:808–23. doi: 10.1002/jcb.10550 12858346

[40] Gong X Xia L Su Z. Friend or foe of innate lymphoid cells in inflammation-associated cardiovascular disease. Immunology. (2021) 162:368–76. doi: 10.1111/imm.13271 PMC7968396 32967038

[41] Yokoyama WM Kim S French AR. The dynamic life of natural killer cells. Annu Rev Immunol. (2004) 22:405–29. doi: 10.1146/annurev.immunol.22.012703.104711 15032583

[42] Jonasson L Backteman K Ernerudh J. Loss of natural killer cell activity in patients with coronary artery disease. Atherosclerosis. (2005) 183:316–21. doi: 10.1016/j.atherosclerosis.2005.03.011 15996672

[43] Hak Ł Myśliwska J Więckiewicz J Szyndler K Trzonkowski P Siebert J. NK cell compartment in patients with coronary heart disease. Immun Ageing I A. (2007) 4:3. doi: 10.1186/1742-4933-4-3 17488493 PMC1878503

[44] Backteman K Ernerudh J Jonasson L. Natural killer (NK) cell deficit in coronary artery disease: no aberrations in phenotype but sustained reduction of NK cells is associated with low-grade inflammation. Clin Exp Immunol. (2014) 175:104–12. doi: 10.1111/cei.12210 PMC3898559 24298947

[45] Lynch LA O'Connell JM Kwasnik AK Cawood TJ O'Farrelly C O'Shea DB. Are natural killer cells protecting the metabolically healthy obese patient? Obes (Silver Spring Md.). (2009) 17:601–5. doi: 10.1111/cei.12210 19238145

[46] Kologrivova I Shtatolkina M Suslova T Ryabov V. Cells of the immune system in cardiac remodeling: main players in resolution of inflammation and repair after myocardial infarction. Front Immunol. (2021) 12:664457. doi: 10.3389/fimmu.2021.664457 33868315 PMC8050340

[47] Li W Lidebjer C Yuan XM Szymanowski A Backteman K Ernerudh J. NK cell apoptosis in coronary artery disease: relation to oxidative stress. Atherosclerosis. (2008) 199:65–72. doi: 10.1016/j.atherosclerosis.2007.10.031 18068708

[48] Huang J Xiao Y Zheng P Zhou W Wang Y Huang G. Distinct neutrophil counts and functions in newly diagnosed type 1 diabetes, latent autoimmune diabetes in adults, and type 2 diabetes. Diabetes/metabolism Res Rev. (2019) 35:e3064. doi: 10.1002/dmrr.3064 30123986

[49] Feng YM Zhao D Zhang N Yu CG Zhang Q Thijs L. Insulin resistance in relation to lipids and inflammation in type-2 diabetic patients and non-diabetic people. PloS One. (2016) 11:e0153171. doi: 10.1371/journal.pone.0153171 27073920 PMC4830613

[50] Särndahl E Bergström I Brodin VP Nijm J Lundqvist Setterud H Jonasson L. Neutrophil activation status in stable coronary artery disease. PloS One. (2007) 2:e1056. doi: 10.1371/journal.pone.0001056 17957240 PMC2020438

[51] Huang X Yang S Zhao Q Chen X Pan J Lai S. Predictive value of non-high-density lipoprotein cholesterol and neutrophil-lymphocyte ratio for coronary artery vulnerable plaques in type 2 diabetes mellitus. Front Cardiovasc Med. (2022) 9:927768. doi: 10.3389/fcvm.2022.927768 35795369 PMC9251121

[52] Kim S Eliot M Koestler DC Wu WC Kelsey KT. Association of neutrophil-to-lymphocyte ratio with mortality and cardiovascular disease in the jackson heart study and modification by the duffy antigen variant. JAMA Cardiol. (2018) 3:455–62. doi: 10.1001/jamacardio.2018.1042 PMC6128503 29801037

[53] Donath MY Dinarello CA Mandrup-Poulsen T. Targeting innate immune mediators in type 1 and type 2 diabetes. Nat Rev Immunol. (2019) 19:734–46. doi: 10.1038/S41577-019-0213-9 31501536

[54] Drucker DJ. Coronavirus infections and type 2 diabetes-shared pathways with therapeutic implications. Endocrine Rev. (2020) 41:bnaa011. doi: 10.1210/endrev/bnaa011 32294179 PMC7184382

[55] Aravindhan V Madhumitha H. Metainflammation in diabetic coronary artery disease: emerging role of innate and adaptive immune responses. J Diabetes Res. (2016) 2016:6264149. doi: 10.1155/2016/6264149 27610390 PMC5004008

[56] Sauer F Riou M Charles AL Meyer A Andres E Geny B. Pathophysiology of heart failure: A role for peripheral blood mononuclear cells mitochondrial dysfunction? J Clin Med. (2022) 11:741. doi: 10.3390/jcm11030741 35160190 PMC8836880

[57] Hartman ML Shirihai OS Holbrook M Xu G Kocherla M Shah A. Relation of mitochondrial oxygen consumption in peripheral blood mononuclear cells to vascular function in type 2 diabetes mellitus. Vasc Med (London England). (2014) 19:67–74. doi: 10.1177/1358863X14521315 PMC3932629 24558030

[58] Laugerette F Vors C Peretti N Michalski MC. Complex links between dietary lipids, endogenous endotoxins and metabolic inflammation. Biochimie. (2011) 93:39–45. doi: 10.1016/j.biochi.2010.04.016 20433893

[59] Donath MY Shoelson SE. Type 2 diabetes as an inflammatory disease. Nat Rev Immunol. (2011) 11:98–107. doi: 10.1038/nri2925 21233852

[60] Virmani R Burke AP Kolodgie F. Morphological characteristics of coronary atherosclerosis in diabetes mellitus. Can J Cardiol. (2006) 22 Suppl B:81B–4B. doi: 10.1016/s0828-282x(06)70991-6 PMC2780829 16498517

[61] Rachinger N Fischer S Böhme I Linck-Paulus L Kuphal S Kappelmann-Fenzl M. Loss of Gene Information: Discrepancies between RNA Sequencing, cDNA Microarray, and qRT-PCR. Int J Mol Sci. (2021) 22:9349. doi: 10.3390/ijms22179349 34502254 PMC8430810

[62] Grätz C Bui MLU Thaqi G Kirchner B Loewe RP Pfaffl MW. Obtaining reliable RT-qPCR results in molecular diagnostics-MIQE goals and pitfalls for transcriptional biomarker discovery. Life (Basel Switzerland). (2022) 12:386. doi: 10.3390/life12030386 35330136 PMC8953338

